# Antioxidant Protects against Increases in Low Molecular Weight Hyaluronan and Inflammation in Asphyxiated Newborn Pigs Resuscitated with 100% Oxygen

**DOI:** 10.1371/journal.pone.0038839

**Published:** 2012-06-11

**Authors:** Helene C. D. Østerholt, Ingrid Dannevig, Myra H. Wyckoff, Jie Liao, Yucel Akgul, Mrithyunjay Ramgopal, Dan S. Mija, Naeun Cheong, Christopher Longoria, Mala Mahendroo, Britt Nakstad, Ola D. Saugstad, Rashmin C. Savani

**Affiliations:** 1 Department of Pediatrics, Akershus University Hospital, Lørenskog, Norway; 2 Institute of Clinical Medicine, University of Oslo, Oslo, Norway; 3 Institute for Surgical Research, Oslo University Hospital – Rikshospitalet, Oslo, Norway; 4 Department of Pediatric Research, Oslo University Hospital – Rikshospitalet, Oslo, Norway; 5 Department of Obstetrics and Gynecology, University of Texas Southwestern Medical Center, Dallas, Texas, United States of America; 6 Divisions of Pulmonary and Vascular Biology and Neonatal-Perinatal Medicine, Department of Pediatrics, University of Texas Southwestern Medical Center, Dallas, Texas, United States of America; University of Florida, United States of America

## Abstract

**Background:**

Newborn resuscitation with 100% oxygen is associated with oxidative-nitrative stresses and inflammation. The mechanisms are unclear. Hyaluronan (HA) is fragmented to low molecular weight (LMW) by oxidative-nitrative stresses and can promote inflammation. We examined the effects of 100% oxygen resuscitation and treatment with the antioxidant, N-acetylcysteine (NAC), on lung 3-nitrotyrosine (3-NT), LMW HA, inflammation, TNFα and IL1ß in a newborn pig model of resuscitation.

**Methods & Principal Findings:**

Newborn pigs (n = 40) were subjected to severe asphyxia, followed by 30 min ventilation with either 21% or 100% oxygen, and were observed for the subsequent 150 minutes in 21% oxygen. One 100% oxygen group was treated with NAC. Serum, bronchoalveolar lavage (BAL), lung sections, and lung tissue were obtained. Asphyxia resulted in profound hypoxia, hypercarbia and metabolic acidosis. In controls, HA staining was in airway subepithelial matrix and no 3-NT staining was seen. At the end of asphyxia, lavage HA decreased, whereas serum HA increased. At 150 minutes after resuscitation, exposure to 100% oxygen was associated with significantly higher BAL HA, increased 3NT staining, and increased fragmentation of lung HA. Lung neutrophil and macrophage contents, and serum TNFα and IL1ß were higher in animals with LMW than those with HMW HA in the lung. Treatment of 100% oxygen animals with NAC blocked nitrative stress, preserved HMW HA, and decreased inflammation. *In vitro,* peroxynitrite was able to fragment HA, and macrophages stimulated with LMW HA increased TNFα and IL1ß expression.

**Conclusions & Significance:**

Compared to 21%, resuscitation with 100% oxygen resulted in increased peroxynitrite, fragmentation of HA, inflammation, as well as TNFα and IL1ß expression. Antioxidant treatment prevented the expression of peroxynitrite, the degradation of HA, and also blocked increases in inflammation and inflammatory cytokines. These findings provide insight into potential mechanisms by which exposure to hyperoxia results in systemic inflammation.

## Introduction

Worldwide, perinatal asphyxia is the single most important cause of brain injury in the newborn, and has consequences that are potentially devastating and lifelong [1]. Although the hypoxic-ischemic insult starts a cascade of events that ultimately may cause cell death and damage to the central nervous system, a systemic inflammatory response that also affects other organ systems including the heart, lungs, liver and kidneys has been described [2]. Despite earlier reports (dating back to 1897) of similar efficacy of either 100% oxygen (O_2_) or room air (21% O_2_), which remained largely ignored, neonatal resuscitation, since its inception, has been carried out using 100% O_2_ [3]. More recently, evidence has accumulated demonstrating that even brief exposure of the lung to hyperoxia is associated with increased mortality, decreased cerebral blood flow and oxidative stress to the kidneys and heart [4], [5], [6]. Indeed, the most recent guidelines for neonatal resuscitation emphasize the use of 21% O_2_ for resuscitation of term newborns [7], and recent reports support a low oxygen strategy for the resuscitation of preterm infants [8], [9]. Examination of the effects of 100% versus 21% O_2_ resuscitation in animal models has been studied most extensively in the pig [10], [11]. Indeed, 100% O_2_ resuscitation is associated with increased inflammatory markers, decreased anti-oxidant capacity, increased oxidative damage to DNA and proteins, and increased evidence of structural damage to the brain [12], [13]. These data suggest that exposure to hyperoxia results in changes that stimulate a systemic inflammatory response that affects multiple organ systems. One potential mechanism could be the formation of reactive oxygen and nitrogen free radicals following exposure to 100% O_2_ [14], [15].

Three groups of ‘reactive species’ have been described, namely reactive oxygen species (ROS), which are partially reduced forms of oxygen (for example, superoxide), reactive nitrogen species (RNS), which are partially reduced nitrogen species (for example, nitric oxide (NO)), and the combination of the two, reactive oxygen and nitrogen species (RONS), which are highly reactive and damaging species (for example, peroxynitrite) [14]. Under physiological conditions, ROS and RNS are produced at low fluxes, with minimal production of RONS, and in a localized manner that result in intracellular signaling [14]. However, with increased fluxes of ROS and RNS, the combination of NO with superoxide to form peroxynitrite occurs at a nearly diffusion limited rate, with a dramatic rise in the production of RONS [16]. In contrast to discrete modifications resulting in cellular signaling, the chemical reactivity of RONS results in non-specific modifications that result in cellular injury and death. Importantly, however, the generation of RONS not only generates toxic species but also reduces the availability of ROS and RNS for signaling functions. Therefore the interaction of ROS and RNS is a two-edged sword that produces both a gain of toxicity and a loss of physiological function. The mechanisms of tissue damage with RONS include disruption of cell membranes (lipid peroxidation), mitochondrial injury, modifications of nucleic acids and DNA scission, activation or inactivation of growth factors, apoptosis and the generation of multiple reactive species that cause further damage [15], [17]. In particular, peroxynitrite causes the nitration of tyrosine residues at the 3-position to produce 3-nitrotyrosine (3-NT) [18]. Nitration has been shown to result in functional alterations of modified proteins, and has been demonstrated in a wide variety of human diseases and in animal models of tissue injury [19], [20], [21].

Hyaluronan (HA), a glycosaminoglycan made up of repeating disaccharide units of glucuronic acid and N-acetyl glucosamine, is an early and important mediator of inflammation [22]. An increased recovery of HA in bronchoalveolar lavage (BAL) has been found in various disease states such as sarcoidosis [23], occupational disorders [24] and Acute Respiratory Distress Syndrome (ARDS) [25], and after acute lung injury as with intratracheal bleomycin instillation in rodents [26], [27], [28]. Further, the increased recovery of HA temporally correlates with an influx of inflammatory cells [29]. HA regulation of inflammation is both dose- and molecular size-dependent. The molecular weight of HA in BAL from injured animals is 200–700 kDa [30], whereas *in situ* lung HA is considerably larger at >10^6^ Dalton. Low molecular weight HA (LMW HA, < ∼500–700 kDa), and HA oligosaccharides (6-30-mer lengths) increase gene expression of proinflammatory chemokines and iNOS in macrophage cell lines as well as in alveolar macrophages from injured rats [31], [32]. The changes in localization, content and size of HA during neonatal asphyxia and resuscitation have not been studied previously.

Since superoxide and peroxynitrite cause chemical fragmentation of HA [33], [34], [35], [36], [37] and LMW HA can stimulate the inflammatory process [29], [38], [39], we hypothesized that exposure to 100% O_2_ during neonatal resuscitation would result in increased peroxynitrite production, fragmentation of HA and an increased inflammatory response. Using a neonatal pig asphyxia model, we examined the early local effects of resuscitation with 100% O_2_ on the lung, and the associated systemic inflammatory response. We here report that exposure of the lung to 100% O_2_ was associated with increased 3-NT and decreased HA staining, and fragmentation of HA in the lung. Both TNFα and IL1ß were increased in 100% O_2_ exposed animals, and, compared to those with HMW HA content, animals with LMW HA content in the lung had significantly more neutrophils and macrophages, as well as higher plasma concentrations of TNFα and IL1ß. Treatment of 100% O_2_-exposed animals with NAC decreased 3NT staining, preserved HMW HA and decreased inflammation and cytokine expression. Furthermore, *in vitro* studies showed that peroxynitrite, and not nitric oxide, fragments HA, and that oligomeric HA stimulates TNFα and IL1ß expression, in a macrophage cell line.

## Results

### Neonatal Pig Resuscitation Model

**Figure 1:**
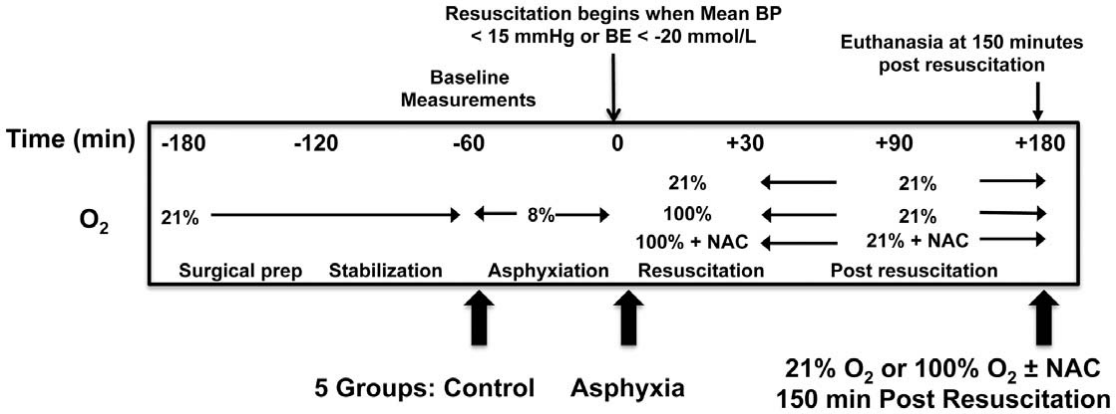
Experimental protocol. After anesthesia, ventilation, and instrumentation followed by 60 min of stabilization, pigs were subjected to asphyxia, followed by either 21% or 100% O_2_ resuscitation for 30 min, and then observed for a further 150 min after resuscitation when all animals were maintained in 21% O_2_. A separate group of animals resuscitated for 30 minutes with 100% O_2_ were treated with the antioxidant N-acetylcysteine (NAC) from the time of the start of resuscitation until the end of the experiment. Samples were obtained from five groups: Control animals were euthanized after undergoing surgical preparation and anesthesia but prior to asphyxia; the asphyxia group was harvested at the end of asphyxia prior to any resuscitation; and three groups of animals resuscitated with either 21% or 100% O_2_ or with 100% O_2_ and given NAC were harvested at 150 min after respective resuscitation strategies.

There were no significant differences across groups with respect to body weight, age, gender and hemoglobin. Baseline hemodynamic and pulmonary parameters, including blood gases, were also not significantly different between the groups (Table 1). The experimental protocol followed, shown in Figure 1, involved the induction of asphyxia by the administration of 8% O_2_. As published by us previously [11], asphyxiated pigs showed profound respiratory/metabolic acidosis, hypoxemia, and hypotension, with similar degrees of compromise at the end of the asphyxia period in both the 21% and 100% O_2_ resuscitation groups (Table 2). As expected, the PaO_2_ of animals at the end of resuscitation was significantly higher in animals resuscitated in 100% O_2_ than those resuscitated in 21% O_2_ (49.1±21.1 kPa vs. 13.0±1.9 kPa, *P*<0.001).

**Table 1:** All pigs were comparable at baseline.

Characteristic	Control (n = 8)	Post-asphyxia (n = 9)	21% O_2_ Resuscitation (n = 8)	100% O_2_ Resuscitation (n = 8)	100% O_2_+ NAC Resuscitation (n = 7)
Weight (g)	1512±189	1585±191	1675±229	1577±274	1309±145
Heart rate (bpm)	149±16	140±13	157±47	147±19	161±20
Mean arterial pressure (mmHg)	55±17	60±14	48±13	50±10	46±6
Arterial pH	7.48±0.09	7.44±0.06	7.47±0.05	7.45±0.10	7.4±0.1
Arterial pCO2 (kPa)	4.9±0.9	5.3±0.6	5.6±0.6	4.8±0.8	3.5±0.4
Arterial pO2 (kPa)	13.5±2.8	13.4±1.9	12.5±1.8	14.3±2.2	11.3±2.4
Base Excess (mmol/L)	3.0±2.8	2.4±5.0	5.7±2.3	0.4±4.5	7.4±3.2
ETCO2 (kPa)	4.1±0.9	4.6±0.5	4.7±0.6	4.0±0.8	5.4±0.7
Lactate (mmol/L)	1.9±0.5	2.0±0.5	2.3±0.8	2.2±0.7	4.4±2.9
Hemoglobin (g)	6.7±1.6	6.9±1.0	6.3±1.5	7.0±0.9	7.4±1.2
Blood Glucose (mmol/L)	6.0±1.5	5.8±1.2	6.3±0.9	5.5±1.0	3.6±1.1

**Table 2:** All pigs were equally asphyxiated.

Characteristic	Control (n = 8)	Post-Asphyxia (n = 9)	21% O_2_ Resuscitation (n = 8)	100% O_2_ Rususcitation (n = 8)	100% O_2_+NAC Rususcitation (n = 7)
Time to asphyxia (min)	–	55±19	63±42	58±25	70±24
Heart rate (bpm)	–	132±33	143±25	160±20	130±25
Mean arterial pressure (mmHg)	–	17±5	15±3	19±4	25±3.2
Arterial pH	–	6.91±0.06	6.92±0.12	6.9±0.09	7.0±0.1
Arterial pCO2 (kPa)	–	9.0±0.3	8.9±1.2	8.9±1.3	8.1±1.3
Arterial pO2 (kPa)	–	4.9±0.4	4.6±0.4	4.6±0.4	4.2±0.3
Lactate (mmol/L)	–	9.4±3.9	11.4±0.9	11.5±3.0	9.7±2.1

### Changes in 3-NT and HA Staining with 100% O_2_ Resuscitation and NAC Treatment

**Figure 2:**
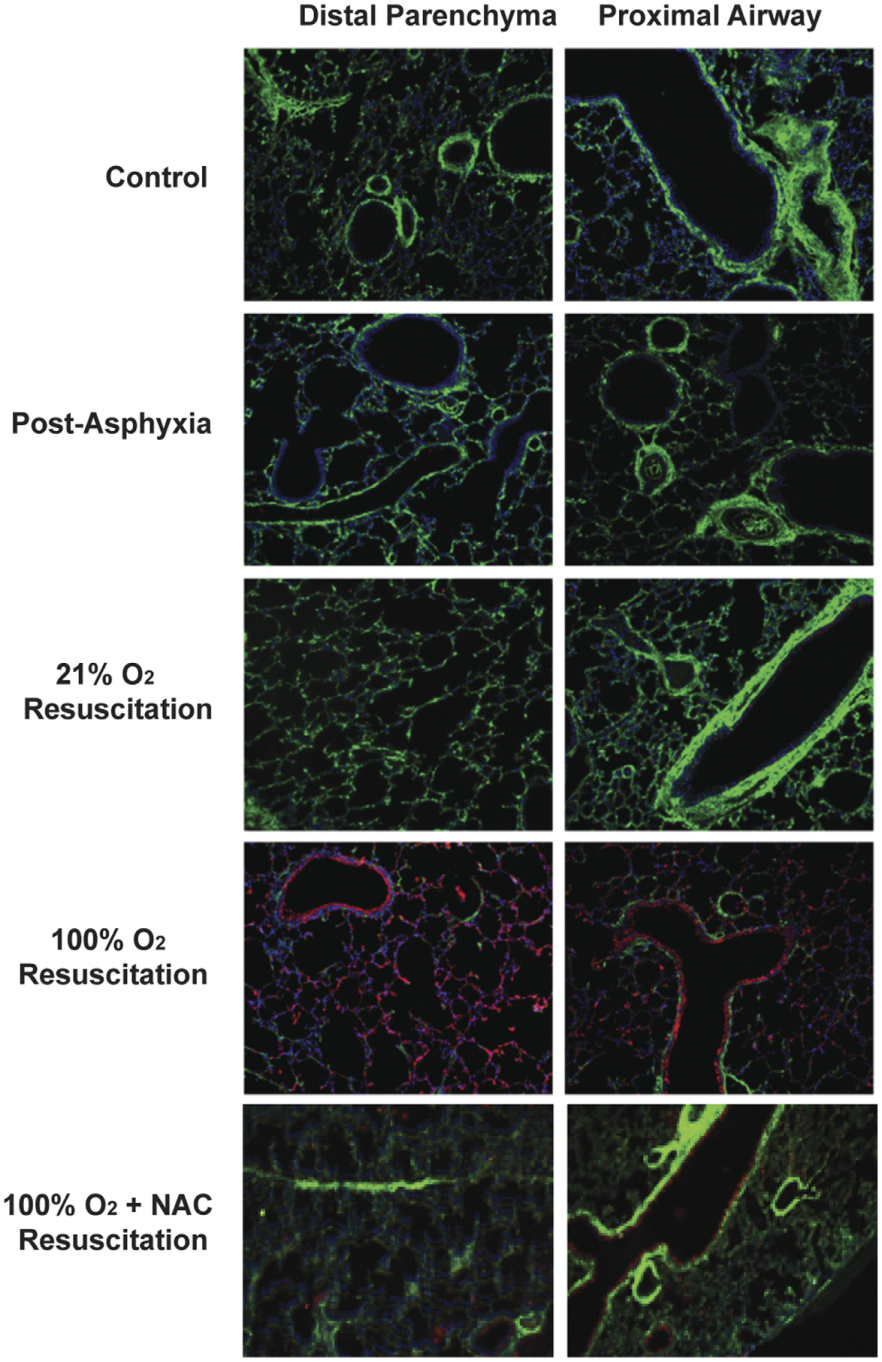
Dual immunostaining for 3-nitrotyrosine and HA. Formalin-fixed, paraffin-embedded sections of inflated lungs were processed for dual label immunofluorescence using an antibody specific for 3-NT to localize peroxynitrite (red), and a biotinylated HA-binding probe to localize HA (green), with DAPI to label nuclei (blue). Control animals had abundant staining for HA in the sub-epithelial matrix around bronchiolar smooth muscle and on the endothelium of blood vessels, with less evident staining in the distal parenchyma and alveoli. At the end of asphyxia, there appeared to be a slight, but generalized decrease in HA staining in both the proximal airway as well as the distal parenchyma. Animals examined 150 minutes after 21% O_2_ resuscitation had HA staining that was not distinguishable from control animals, and had no staining for 3-NT. However, 150 minutes after 100% O_2_ exposure, little to no HA staining and an increase in 3-NT staining was observed throughout the lung. Animals resuscitated with 100% O_2_ and given NAC had little 3-NT staining and had HA staining similar to that of animals resuscitated with 21% O_2_.

Formalin-fixed, paraffin-embedded sections of inflated lungs were processed for dual label immunofluorescence using an antibody specific for 3-NT to localize peroxynitrite (red), and a biotinylated HA-binding probe to localize HA (green), with DAPI to label nuclei (blue) as described in Materials and Methods. Images were obtained from both the distal parenchyma and the proximal airway. Control animals had abundant staining for HA in the sub-epithelial matrix around bronchiolar smooth muscle and on the endothelium of blood vessels, with less evident staining in the distal parenchyma and alveoli (Fig. 2). Animals examined 150 minutes after the 30-minutes exposure to 21% O_2_ resuscitation had HA staining that was not distinguishable from control animals, and had no staining for 3-NT (Fig. 2). However, 150 minutes after the 30-minute exposure to 100% O_2_, little to no HA staining and a significant increase in 3-NT staining was observed throughout the lung (Fig. 2). Interestingly, animals resuscitated with 100% O_2_ and treated with NAC had substantially less 3-NT staining, and HA staining that was similar to control animals. These data demonstrate that 3-NT is only evident after hyperoxia exposure and is associated with decreased HA in the lung, and that treatment with the antioxidant decreases 3-NT and preserves HA staining.

### Changes in BAL and Serum HA with 100% O_2_ Resuscitation and NAC Treatment

**Figure 3:**
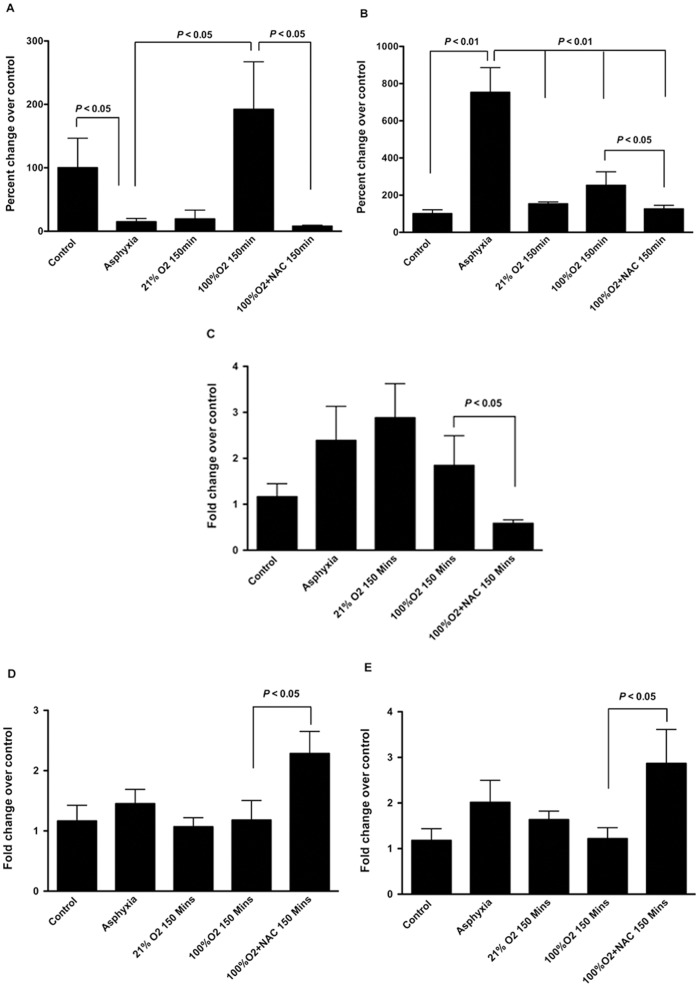
Hyaluronan content in bronchoalveolar lavage and serum, and expression of enzymes regulating hyaluronan synthesis and degradation. HA content was determined in BAL (A) and in serum (B). At the end of asphyxia, the HA content of BAL decreased and that in the serum increased. Resuscitation with 21% O_2_ did not increase HA either in the BAL or in the serum. However, BAL HA was significantly increased in animals resuscitated with 100% O_2_, and this increase was completely inhibited by treatment with NAC (A). The changes in hyaluronan synthase 1 (*has1*), *has2* and *has3*, as well as hyaluronidase 1 (*hyal1*) and 2 (*hyal2*) were determined using quantitative RT-PCR. There were no changes in *has1* and *has 3* expression (data not shown). Expression of *has2* (C) showed a trend to increased expression after asphyxia and 21% O_2_ resuscitation. However, treatment with NAC significantly inhibited has2 expression compared to 100% O_2_ resuscitation alone (C). The expression of *hyal1* (D) and *hyal2* (E) remained unchanged except that 100% O_2_ exposed animals treated with NAC showed significantly increased expression of both hyaluronidases.

We next determined the content of HA in both BAL and serum under the various experimental conditions. Data were compared as a percent of control animals. BAL HA content decreased and serum HA increased with asphyxia (Fig. 3A and B). At 150 minutes after the end of the resuscitation period, animals resuscitated in 100% O_2_ had significantly higher BAL HA concentrations than animals resuscitated in 21% O_2_ (Fig. 3A). Animals exposed to 100% O_2_ and treated with NAC did not show this later increase in BAL HA (Fig. 3A). While serum HA did not show a significant rise at 150 minutes in animals exposed to 100% O_2_, treatment with NAC decreased serum HA concentration (Fig. 3B). In order to determine the balance of HA synthesis and enzymatic degradation of HA, we determined the lung mRNA contents for the three HA synthase genes (*has1*, *2* and *3*) as well as two hyaluronidases (*hyal1* and *hyal2*). No significant differences were noted in *has1* and *has3* expression under any condition (data not shown). The expression of *has2* was significantly decreased (Fig. 3C), and the expression of *hyal1* and *hyal2* was significantly increased in animals treated with NAC (Fig. 3D and E), suggesting that the net result would be less HA with anti-oxidant treatment.

**Figure 4:**
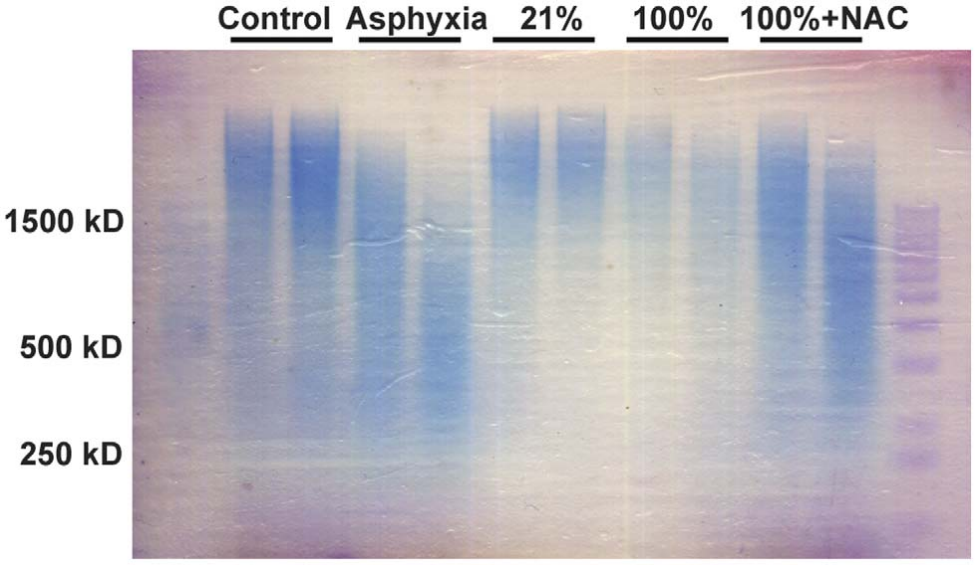
HA size determination. HA size was determined in lung tissue and two representative samples per group are shown in this gel. Control animals had HMW HA and asphyxia was associated with some degradation of HA. Resuscitation with 21% O_2_ was associated with HMW HA where as resuscitation with 100% O_2_ showed marked degradation of HA. Interestingly, treatment of 100% O_2_ resuscitated animals with NAC was associated with a preservation of HMW HA.

Pig lungs were then processed to determine the molecular size of HA as described in Materials and Methods (Fig. 4). Control animals had largely HMW HA, whereas pigs at the end of the asphyxia period had an intermediate size HA. Interestingly, 21% O_2_ exposed animals demonstrated HMW HA suggesting increased synthesis, whereas those exposed to 100% O_2_ showed almost completely degraded HA (Fig. 4). Interestingly, 100% O_2_ exposed animals treated with NAC showed substantial preservation of HMW HA (Fig. 4). Given the decreased *has2* and increased *hyal1/2* expression (Fig. 3), it is most likely that the decreased oxidative and nitrative stress noted with NAC treatment (Fig. 2) prevented the fragmentation of HA. We next determined the proportion of animals with LMW HA in the lung in the various groups studied (Table 3). Interestingly, control animals had almost exclusively HMW HA in the lung, whereas pigs at the end of asphyxia had exclusively LMW HA. Animals resuscitated in 21% O_2_ returned to HMW HA whereas those resuscitated in 100% O_2_ continued to have LMW HA in the lung. Animals treated with NAC had mostly HMW HA in their lungs. Collectively, these data suggest that exposure to 100% O_2_ was associated with an elevated content of LMW HA, and that antioxidant treatment was associated with a shift to HMW HA.

**Table 3:** Proportions of pigs with LMW HA after asphyxia and 21% vs. 100% O_2_ resuscitation.

Groups	HMW (n)	LMW (n)	% LMW
Control (n = 8)	7	1	14%
Post-Asphyxia (n = 9)	0	9	100%
21% O_2_ Resuscitation (n = 8)	6	2	25%
100% O_2_ Resuscitation (n = 8)	3	5	63%
100% O_2_+ NAC Resuscitation (n = 7)	5	2	29%

The majority of control animals had HMW HA in the lung. At end of asphyxia, pigs had exclusively LMW HA, which recovered to a preponderance of HMW HA with 21% O_2_ resuscitation. Exposure to 100% O_2_ resuscitation was associated with a change from HMW to LMW HA. Treatment with NAC showed a protection of HMW HA. These data show that asphyxia and exposure to 100% O_2_ is associated with fragmentation of HA in the lung and that antioxidant treatment protects against this degradation *in vivo*.

### Increased Lung Neutrophil and Macrophage Contents in Animals with LMW HA are Inhibited by Antioxidant Treatment

**Figure 5:**
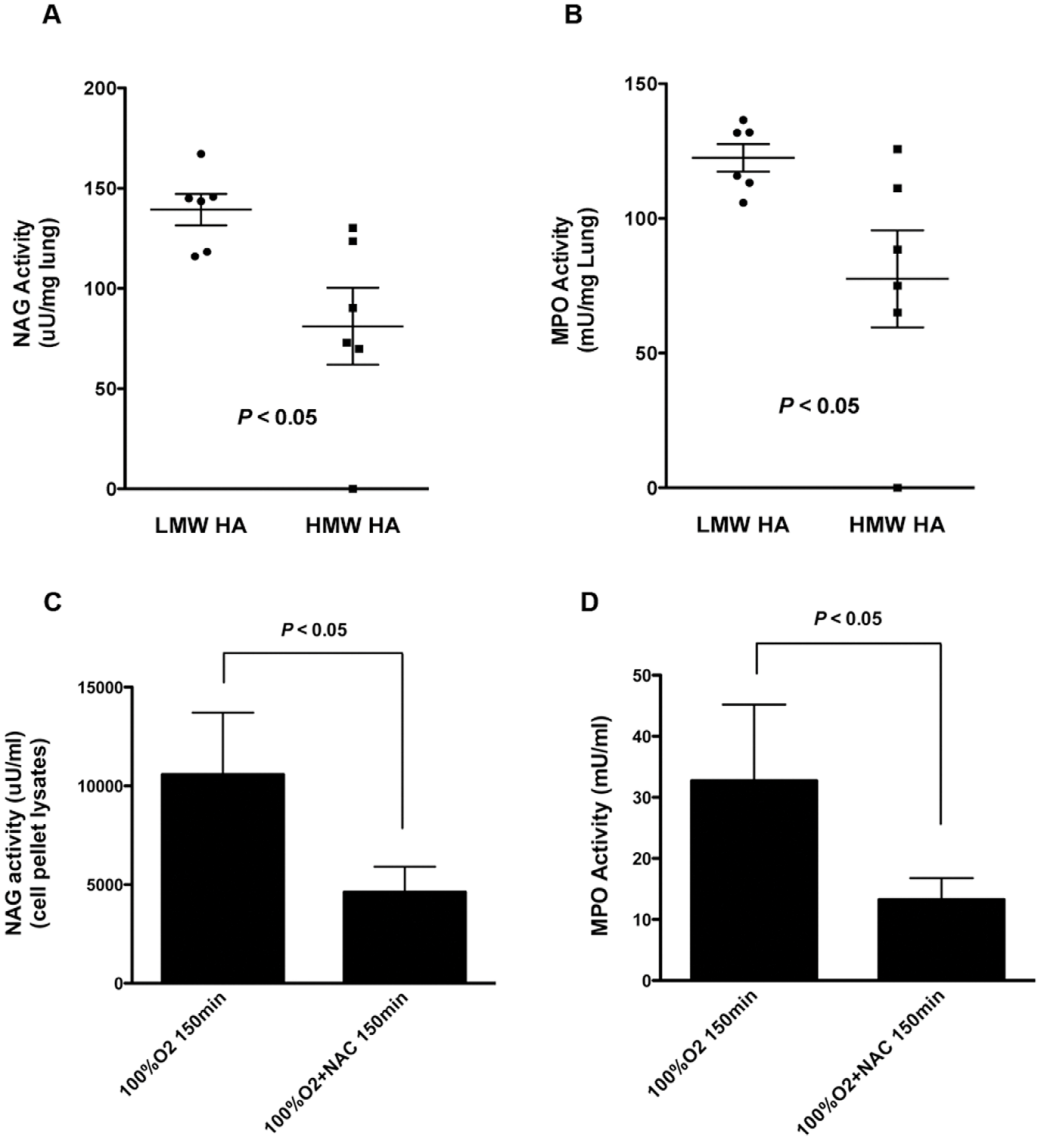
Lung myeloperoxidase and N-acetylglucosaminidase activities as measures of neutrophil and macrophage contents respectively. Macrophage content was determined by NAG activity and neutrophil content was determined by MPO activity as described previously [82] and in Materials and Methods. Data were segregated according to the molecular size of HA found in the lung and plotted as box and whisker plots with outliers shown as additional dots. Both NAG (A) and MPO (B) activities were significantly higher in animals that had LMW HA. Newborn pigs resuscitated with 100% O_2_ and treated with NAC had significantly lower NAG (C) and MPO (D) activities than those without NAC treatment, suggesting that antioxidant treatment decreases the inflammatory response to resuscitation with hyperoxia.

We next determined myeloperoxidase (MPO) and N-acetyl glucosaminiadase (NAG) activities of lung as measures of neutrophil and macrophage contents respectively (Fig. 5). Data were segregated according to the molecular size of lung HA and plotted as interquartile ranges. Animals with LMW HA had significantly higher MPO and NAG activities confirming an increased accumulation of neutrophils and macrophages in animals that had LMW HA in the lung (Fig. 5A and B). Animals exposed to 100% O_2_ and treated with NAC had significantly lower MPO and NAG activities as compared to animals exposed to 100% O_2_ alone (Fig. 5C and D). These data suggest that antioxidant treatment was associated with decreased inflammation after 100% O_2_ resuscitation.

Since inflammation is often associated with edema, we also determined whether any differences in water content existed between the various groups. No significant differences in water content were found in any group (data not shown).

### Increased Serum TNFα and IL1ß Concentrations in Animals with LMW HA are Inhibited by Antioxidant Treatment

**Figure 6:**
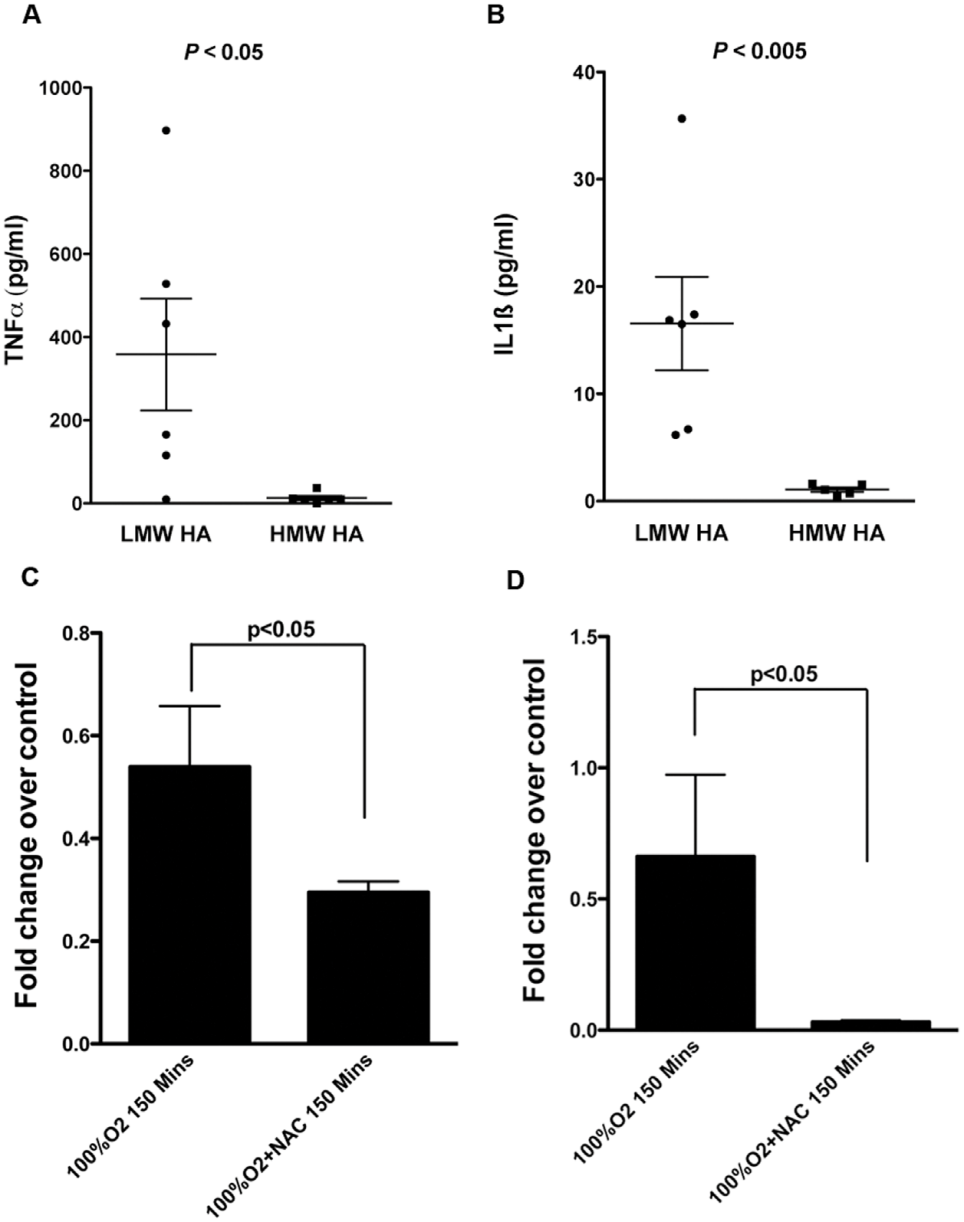
Changes in BAL TNFα and IL1ß concentrations. The BAL contents of TNFα (A) and IL1ß (B) were determined by ELISA were also segregated according to the molecular size of HA. Data are presented as box and whisker plots of 25^th^ and 75^th^ percentiles showing outliers as additional dots. Both inflammatory markers were significantly higher in the animals that had LMW HA in the lung. Newborn pigs resuscitated with 100% O_2_ and treated with NAC had significantly lower TNFα (C) and IL1ß (D) than those without NAC treatment, suggesting that antioxidant treatment decreases the inflammatory response to resuscitation with hyperoxia.

Serum TNFα and IL1ß contents were determined using commercially available ELISA kits. Data were again segregated according to the molecular size of HA in the lung and presented as interquartile ranges. Animals with LMW HA had significantly higher TNFα and IL1ß concentrations (Fig. 6A and B). Treatment of 100% O_2_ exposed animals with NAC resulted in significantly lower TNFα and IL1ß concentrations (Fig. 6C and D). Collectively, these data suggest that hyperoxia exposure to the lung results in increased serum TNFα and IL1ß concentrations in association with LMW HA, and that NAC treatment is associated with lower cytokine levels.

### Peroxynitrite, but Not Nitric Oxide, Fragments HMW HA to LMW HA In vitro

**Figure 7:**
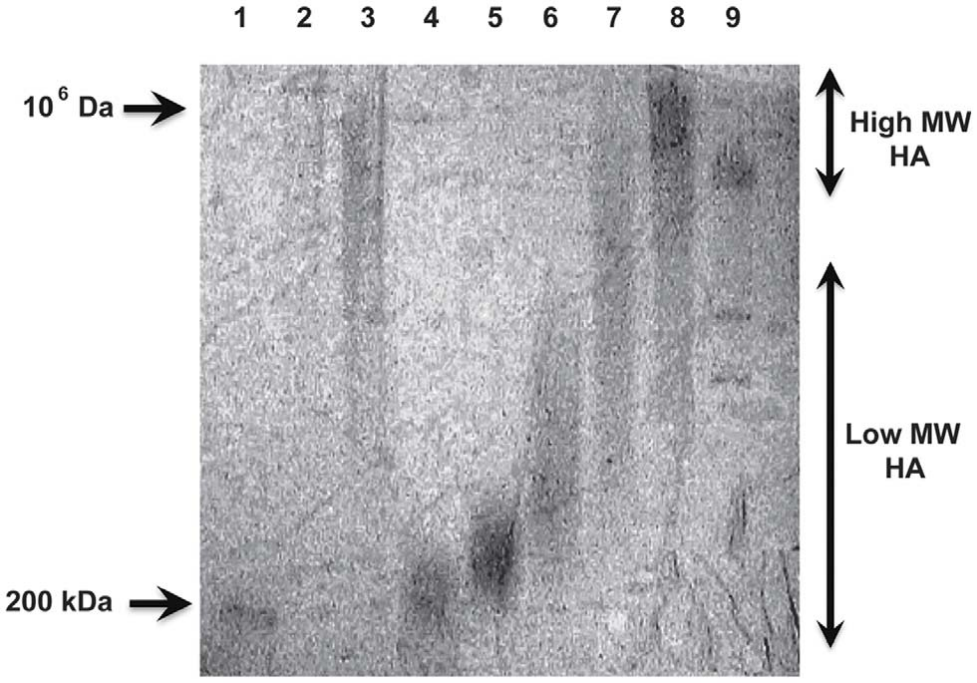
Fragmentation of HA by peroxynitrite and stimulation of TNFα and IL1ß by oligomeric HA in vitro. HMW HA (Healon™, 1×106 Da, Lane 2) was exposed to 100 µM 3-morpholinosydnonimine (SIN-1), a compound that spontaneously releases NO and superoxide to generate peroxynitrite. Exposure of HMW HA to SIN-1 results in degradation to LMW HA (300–500 kDa, Lane 6). Exposure to SIN-1 in the presence of 600 mU/ml superoxide dismutase (SOD) partially protects this degradation (Lane 7). Exposure to 300 µM PAPANOATE, a pure NO donor, does not degrade Healon™ (Lane 8). In addition, treatment of Healon™ with either 1 U/ml Streptomyces hyaluronidase at 60°C for 2 hours (Lane 4) or sonication for 2 minutes (Lane 5) results in formation of LMW HA. Just heating HMW HA at 60°C in the absence of hyaluronidase did not degrade HA in the same manner as in the presence of enzyme (Lane 2). Lane 1 has 200 kDa HA (ICN) and Lane 9 contains Hind III digested DNA makers.

In order to confirm the specificity of the fragmentation of HA by peroxynitrite, we exposed HMW HA (Healon™, 1×10^6^ Da) to 100 µM 3-morpholinosydnonimine (SIN-1), a compound that spontaneously decomposes to release NO and superoxide to generate peroxynitrite at pH 7.4 (Fig. 7). Exposure of HMW HA to SIN-1 resulted in degradation to LMW HA (300–500 kDa). Exposure to SIN-1 in the presence of superoxide dismutase (SOD) partially protected this degradation, whereas exposure to PAPANOATE, a pure NO donor, did not degrade Healon™. In addition, treatment of HMW HA with either *Streptomyces* hyaluronidase at 60°C for 2 hours or sonication for 2 minutes resulted in formation of LMW HA. These data show that superoxide and peroxynitrite, and not nitric oxide itself, fragment HMW to LMW HA.

### Oligomeric HA Stimulates TNFα and IL1ß Expression in Macrophages In vitro

**Figure 8:**
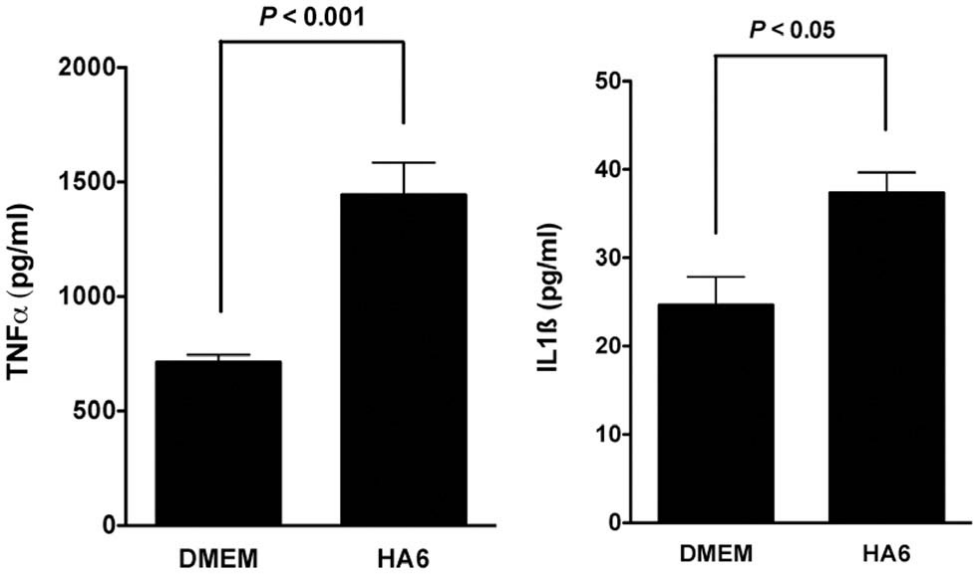
Stimulation of TNFα and IL1ß production by a 6mer HA oligosaccharide. RAW264.7 murine macrophages (1×10^6^) were stimulated with a 10 µg/ml of 6-mer HA oligosaccharide *in vitro*. Supernatant IL1ß and TNFα were measured using standard ELISA. HA6 significantly stimulated the expression of both IL1ß and TNFα in the macrophage cell line, thereby confirming that LMW HA can stimulate the expression of inflammatory cytokines in macrophages.

In order to determine the effects of LMW HA on TNFα and IL1ß expression, we stimulated the murine macrophage cell line RAW264.7 with a six-sugar length HA oligosaccharide (HA6) that was confirmed to be free of endotoxin, nucleic acid and protein. HA6 (10 µg/ml) significantly stimulated the production of TNFα and IL1ß (Fig. 8). Collectively, these data support the hypothesis that the presence of peroxynitrite fragments HMW to LMW HA and that LMW HA stimulates the production of TNFα and IL1ß in macrophages.

## Discussion

In this study, we demonstrate that exposure to 100% and not 21% O_2_ during resuscitation is associated with increased peroxynitrite and LMW HA content in the lung. Interestingly, increased inflammatory cell accumulation and elaboration of TNFα and IL1ß was noted in animals that had LMW HA in the lung. Treatment with the antioxidant, N-acetylcysteine (NAC), was associated with decreased peroxynitrite, preserved HMW HA and less inflammatory cell accumulation and cytokine expression. Further, in cultured murine macrophages, peroxynitrite was able to degrade HMW to LMW HA, and oligomeric HA was able to stimulate TNFα and IL1ß production. These data suggest potential mechanisms by which oxygen exposure during resuscitation could fragment HMW to LMW HA and stimulate an inflammatory response.

The use of oxygen in human resuscitation was attempted within 5 years of its discovery in 1772 [3]. Despite later descriptions of the possibility of adequate resuscitation with room air, and pre-clinical studies that demonstrated the potential harmful effects of oxygen radicals, the practice of 100% O_2_ resuscitation continued. The Resair2 study, published in 1998 [40], demonstrated that newborn infants could be resuscitated effectively with 21% O_2_, and resulted in a faster time to first breath and cry. Further, 21% O_2_ resuscitation was not associated with adverse effects in neurodevelopmental outcome [41]. Multiple other clinical trials have now been conducted that demonstrate that 21% O_2_ resuscitation of term infants is as efficient as 100% O_2_ and is associated with decreased mortality [4]. However, the mechanisms by which 100% O_2_ exposure to the lung causes harm have not been fully examined.

The best-studied model of neonatal resuscitation to date is the newborn pig [10]. The maturation of the pig brain is similar to that of a term human infant, and the newborn pig size allows the use of intensive care equipment used for babies. A number of studies have demonstrated increased accumulation of inflammatory cells in the lung, elevated lung inflammatory markers, and decreased lung compliance with 100% O_2_ exposure during resuscitation [42], [43]. Additionally, this level of O_2_ exposure is associated with decreased anti-oxidant capacity, increased oxidative damage to DNA and proteins, increased apoptosis and evidence of structural damage to the brain [13]. At the same time, it is important to note the limitations of the newborn pig model used in these studies. While useful as a controlled experimental model to determine the mechanisms involved, it does not exactly reproduce the clinical setting. For example, there is no fetal to neonatal transition, the animals receive a long exposure (30 minutes) to 100% O_2_, and the animals are sacrificed at very short intervals from the injury.

Multiple studies in animal models indicate that superoxide and nitric oxide (NO) participate in hyperoxic lung injury [44], [45], [46]. High levels of NO, such as those present with inflammation, react with superoxide to yield higher oxides of nitrogen, in particular peroxynitrite [47]. Peroxynitrite, an oxidant capable of damaging alveolar epithelium and pulmonary surfactant [48], [49], reacts with proteins to form 3-nitrotyrosine, thereby altering protein function [50], [51], [52]. Indeed, nitration of myosin light chain 2 in the hearts of asphyxiated pigs is associated with cardiac dysfunction [53]. It is important to remember that exposure to hyperoxia provokes a systemic hyperoxic challenge with effects on multiple organ systems. Indeed, the formation of oxygen and nitrogen free radicals influences the development of pulmonary hypertension [54]. In addition, N-acetyl-glucosaminidase, the macrophage marker used in the current studies, is also found in the liver and kidneys, and is significantly increased in babies with asphyxia that have been resuscitated with 100% O_2_ [6]. Cheung et al. have examined the effects of antioxidant treatment in this model of resuscitation. Treatment with NAC, which blocks the production of superoxide and peroxynitrite, results in decreased oxidative stress, improved hemodynamics and tissue perfusion, and decreased platelet aggregation after hypoxia-reoxygenation [55], [56], [57], [58], [59], [60]. However, the mechanisms by which oxidative and nitrative stresses promote inflammation have not been fully defined.

Hyaluronan (HA) is an early and important mediator of inflammation [22], [39]. HA regulation of inflammation is both dose- and molecular size-dependent. HA, at doses of 1 mg/ml or greater, inhibits inflammatory cell chemotaxis [61], phagocytosis and respiratory burst activity [62], as well as elastase release [63]. HA also acts as an anti-inflammatory and anti-fibrotic agent in rheumatoid and osteoarthritis [64], and in repair of tympanic membrane perforations [65]. In addition, HA accelerates cutaneous wound healing [66] and reduces adhesion formation after intra-abdominal surgery [67]. On the other hand, at lower concentrations and at lower molecular weights, HA promotes monocyte maturation into macrophages as measured by production of insulin-like growth factor-1 [68], and HA is greatly increased during inflammatory conditions such as myocardial infarction [69], arthritis [70] and during transplant rejection [71]. Furthermore, removal of HA with early treatment of myocardial infarction with hyaluronidase results in reduced myocardial fibrosis and infarct size [72]. We previously demonstrated that alveolar macrophages from bleomycin-injured animals are more motile than those from control animals and that HA-binding peptide is able to completely inhibit this increased motility [29]. Further, systemic administration of HA-binding peptide to animals prior to injury resulted in decreased macrophage accumulation and fibrosis [29]. These data suggest that HA is upstream of and critical for the inflammatory response to lung injury. HMW HA can be fragmented to LMW HA chemically by exposure to superoxide and peroxynitrite [35], [73]. Our findings that treatment with NAC results in decreased 3-NT and preservation of HMW HA, and that the balance of HA synthetic and degrading enzymes favors HA degradation, strongly suggests that HA fragmentation is largely due to chemical rather than enzymatic processes. Thus, treatment with NAC shifts the lung HA environment from a pro-inflammatory to an anti-inflammatory predominance. This is confirmed by our findings that NAC treatment was associated with decreased inflammation and cytokine elaboration.

We also showed that superoxide and peroxynitrite generation by SIN-1 resulted in fragmentation of HMW HA *in vitro*. Importantly, however, a pure nitric oxide donor failed to fragment HA, suggesting that it is the combination of superoxide and nitric oxide that is relevant for the observation. Indeed, administration of high concentrations of NO in conjunction with hyperoxia is harmful to the lung, whereas lower concentrations are beneficial [74]. In addition, studies in extracellular superoxide dismutase (EC-SOD) knockout mice demonstrate that this anti-oxidant enzyme prevents the effects of superoxide and peroxynitrite on fragmentation of HA and limits inflammatory responses to lung injury [75].

The stimulation of cytokine production by HA has been demonstrated previously. However, the mechanisms by which this occurs are unclear. It has been postulated that CD44, a type 1 receptor for HA, complexes with TLR4 to mediate signals that activate the innate immune system [22], [39], [76], [77]. Whether this complex also regulates the production of TNFα and IL1ß after 100% O_2_ resuscitation and whether other cytokines are also affected in a similar manner have yet to be investigated.

**Figure 9:**
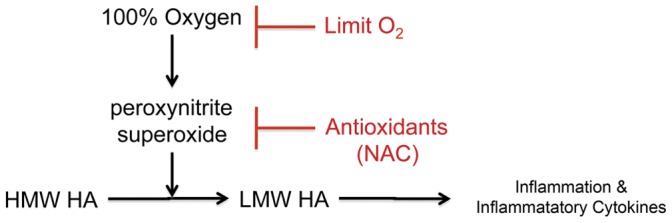
Overall model of the role of oxidative and nitrative stresses and LMW HA in asphyxia and hyperoxia-stimulated inflammation. Exposure to 100% oxygen in asphyxiated newborn pigs results in the production of superoxide and peroxynitrite that cause the fragmentation of HMW to LMW HA. LMW HA, in turn stimulates inflammatory cytokine expression in macrophages and promotes inflammation. Strategies to prevent the formation of LMW HA, such as limiting oxygen exposure or treatment with antioxidants (the current work) will result in decreased inflammation. This model predicts that direct blockade of LMW HA should also achieve the same result.

In summary, we here demonstrate that resuscitation of neonatal pigs with 100% O_2_ is associated with increased peroxynitrite, fragmentation of HA and increased TNFα and IL1ß production. Treatment with the antioxidant NAC is associated with decreased peroxynitrite, preservation of HMW HA and decreased cytokine production. The current findings provide proof-of-concept that antioxidant therapy has the potential to limit damage in situations where 100% O_2_ is used during resuscitation. However, the level of O_2_ exposure that results in the observations made in this report are yet to be defined in the newborn pig model, and the efficacy of antioxidant therapy during human resuscitation will need extensive study. Indeed, with the use of 21% O_2_ resuscitation for term infants, there should be decreased need for the use of antioxidant therapy. The model that we have developed for the mechanisms explored by the current studies is shown in Figure 9. We speculate that limiting the use of 100% O_2_ during resuscitation, or blockade of the effects of LMW HA, for example by using HA-binding peptide, will limit the systemic inflammatory response and potentially decrease end organ damage in neonates suffering asphyxia.

## Materials and Methods

### Ethics Statement

The Department of Comparative Medicine, Oslo University Hospital (Protocol # 37/06) and the University of Texas Southwestern Medical Center Institutional Animal Care and Utilization Committee (Protocol # 2011-0002) approved the experimental protocol. Animals were cared for and handled in accordance with the Norwegian Council for Animal Research, as well as European and NIH Guidelines for Use of Experimental Animals, by certified FELASA (Federation of European Laboratory Animals Science Association) and UT Southwestern researchers.

### Surgical Preparation and Anesthesia

Forty newborn Noroc (LYxLD) pigs, 12–36 h of age, Hb ≥5 g/dL, and in good general condition were included in the study. The pigs were anesthetized, orally intubated, ventilated, and surgically prepared as described by Andresen et al. [78]. Briefly, after anesthesia induced by Sevofluran 5% (Sevorane, Abbott), intravenous pentobarbital and fentanyl were administered, and anesthesia was continued with fentanyl and midazolam infusions. A continuous intravenous infusion of 0.7% saline and 1.25% glucose was given throughout the experiment. A tracheotomy was performed and animals were ventilated using a pressure-controlled ventilator to achieve normal ventilation (PaCO_2_ 4.5–6.0 kPa, O_2_ saturations >90% & tidal volume 8–15 ml/kg). The left femoral artery and vein were cannulated with polyethylene catheters. Rectal temperature was maintained between 38.5 and 40°C with a heating blanket and radiant heating lamp.

### Experimental Protocol

After 60 min of stabilization, the pigs were subjected to global hypoxia, followed by either 21% or 100% O_2_ resuscitation for 30 min, and then observed for a further 150 min after resuscitation when all pigs were maintained in 21% O_2_. In separate experiments, as described previously [59], [60], an additional group of animals exposed to 30 minutes of 100% O_2_ resuscitation were treated with a bolus of 150 mg/kg N-acetylcysteine (NAC) starting at the onset of 100% O_2_ resuscitation followed by a 20 mg/kg/h infusion for the duration of the experiment. Samples were obtained from five groups: Control animals were euthanized after undergoing surgical preparation and anesthesia but prior to asphyxia; the post asphyxia group was harvested at the end of asphyxia prior to any resuscitation; and three groups of animals were harvested at 150 minutes after the 30 minute exposure to either 21% O_2_ resuscitation, 100% O_2_ resuscitation, or 100% O_2_ resuscitation with NAC treatment.

For asphyxiated groups, hypoxemia was achieved by ventilation with a gas mixture of 8% O_2_ in N_2_ until either mean arterial blood pressure decreased to 15 mm Hg or base excess (BE) reached -20 mM/L. CO_2_ was added during hypoxemia aiming at a PaCO_2_ of 8.0–9.5 kPa, to imitate perinatal asphyxia. Before resuscitation, hypoxic pigs were divided into experimental groups. Resuscitation was performed for 30 min with 21% or 100% O_2_. In separate experiments, a 100% O_2_ group was also treated with NAC from the start of resuscitation. After the initial 30 minutes of resuscitation, the pigs all received 21% O_2_, were maintained in normocapnia (PaCO_2_ 4.5–5.5 kPa), and were observed for a further 150 min. Although no animals died during the experiments, two animals observed for 150 minutes following resuscitation (one in the 21% O_2_ group and one in the 100% O_2_ group) needed short intervals of bag & mask ventilation during this observation period. Three animals that required vasoactive drugs during the studies were excluded from the experimental analysis. At the end of each observation time, the animals were given an overdose of pentobarbital (150 mg/kg IV). Bronchoalveolar lavage and plasma samples were obtained, and tissues were quickly removed, snap frozen in liquid nitrogen, and stored at -70°C until subsequent analysis.

### Antibodies & Other Reagents

Polyclonal anti-3-nitrotyrosine antibody was obtained from Cell Signaling Technology (Danvers, MA). Biotinylated HA-Binding Protein (bHABP, Seikagaku Corporation, Tokyo, Japan), a probe that binds all forms of HA six saccharide units or greater, is the biotinylated HA-binding region of aggrecan extracted from nasal bovine cartilage as described by Ripellino et al. [79]. Horseradish peroxidase-conjugated goat anti-rabbit IgG was purchased from Zymed Laboratories (San Francisco, CA). Healon is a pure HMW HA used for ophthalmologic surgery. HA6, a six saccharide HA, was the kind gift of Seikagaku Corporation, and was verified to be free of endotoxin, protein and nucleic acid. All other reagents were purchased from Sigma-Aldrich (St. Louis, MO) unless otherwise noted.

### Immunostaining for 3-nitrotyrosine and HA

Indirect dual immunofluorescence for 3-NT and HA was performed on 5 µm paraffin-embedded sections. After paraffin removal and rehydration, endogenous fluorescence was blocked with 0.1% sodium borohydride for 10 minutes followed by incubation with fresh 1M glycine in PBS for 1 hour. Nonspecific binding was blocked by incubation with 100% goat serum for 60 minutes at 4°C. Sections were then incubated with 3-NT antibody (1:500 dilution) overnight at 4°C. Incubations without primary antibody or with pre-immune rabbit IgG were used as negative controls. After washing in PBS with 0.02% Na azide, HA was localized by incubating sections overnight with the biotinylated HA binding region of the aggrecan (bHABP 1:200 dilution; Seikagaku Corporation, Tokyo, Japan). A solution of preincubated HA:bHABP 3:1 was used as a negative control. Texas Red-conjugated goat anti-rabbit IgG and FITC-conjugated streptavidin (each 1:5,000 dilution) were used as secondary probes, exposed for 3 hours, and DAPI was added to localize nuclei. The slides were then washed and mounted with Fluoromount-G (Southern Biotechnology, Birmingham, AL). Labeled sections were visualized using an inverted Nikon TE 100 microscope. Simultaneous wavelength scanning allowed superimposition of fluorescent labeling with FITC and Texas red fluorophores at wavelengths of 488 and 568 nm, respectively. Overlays were accomplished using Metamorph software (Universal Imaging, Downingtown, PA).

### ELISA-Like Assay for Hyaluronic Acid (HA)

BAL samples were assayed for HA content by an ELISA-like assay as previously described [29]. This ELISA measures the competition of HA present in the sample verses HA coated on a 96-well plate for binding to a biotinylated HA-binding protein (bHABP Seikagaku, Japan). Briefly, 60 µl of sample or Healon standard (Pharmacia, Sweden) were loaded onto non-fat dry milk (NFDM)-blocked Covalink-NH 96-Microwell plates (Nunc, Fisher Corp.) after overnight protease digestion. After addition of 60 µl bHABP to each well and incubation at 37°C for 1 hour, 100 µl of the sample-bHABP incubation solution were transferred to a HA-coated Covalink-plate and incubated for 1 hour at 37°C to allow to competitive binding (0.2 mg/ml HA, ICN Inc.). HA-binding was detected by an avidin-biotin complex (ABC) reagent (Vectastain) and o-phenylenediamine (Sigma). The change in absorbance at 450 nm after a 15-minute incubation was measured.

### Tissue Water Content and Determination of HA Size

Wet and dry weights of lung tissues were taken before and after lyophilization, respectively. Tissue water content was calculated by subtracting dry and wet weight ratio from 1. Lyophilized tissues were digested in 100 mM ammonium acetate with 0.0005% phenol red (pH 7.0) containing 0.25 mg/mL proteinase-K (Roche, Indianapolis, IN) for 4 h at 60°C with occasional vortexing. Proteinase-K was inactivated by boiling, and undigested tissues were pelleted by centrifugation at 16000×g. An aliquot of 100 µl supernatant equal to 5 mg dry weight of digested lung was processed for HA molecular weight determination as described elsewhere with minor modifications [80]. To remove DNA and RNA from tissue extracts, tissues were treated with 3 µl of DNase (Ambion, Austin, Texas) and 3 µl of RNaseA (1.28 mg/ml, Roche, Indianapolis, IN), respectively, for 5 h at 37°C. Samples were boiled to inactivate enzymes and HA was precipitated in 80% ethanol at −20°C overnight. Following centrifugation, pellets were resuspended in 16 µl of Tris-Na Acetate - EDTA, pH.7.9 and 4 µl loading buffer (0.2% Bromophenol Blue, 1 ml TAE buffer and 8.5 ml glycerol). Samples were run on a 1% agarose gel (Seakem HGT Cambrex, Rockland, ME) made in TAE buffer. The gel was pre-run for ∼2 h at 80V prior to loading samples and HA size standards (Hyalose, Oklahoma City, OK). After electrophoresis at 80 V, the gel was equilibrated in water for 48 h followed by incubation in 30% ethanol for 30 min. The gel was then stained with 0.01 mg/ml Stains-All solution (Sigma, St Louis, MO) in 30% ethanol overnight in the dark. Gel was destained in water until bands were visualized before scanning.

### Quantitative RT-PCR

Total RNA was extracted from lung tissues using RNeasy Plus Mini Kit (QIAGEN Inc., Valencia, CA, USA), following the manufacturer’s instructions. Extracted RNA concentration of each sample was quantified using a NanoDrop ND-1000 Spectrophotometer (Thermo Fisher Scientific, Wilmington, DE, USA). Reverse transcription was performed with 1 µg of total RNA in a 20 µl volume using iScript cDNA synthesis kit (BIO-RAD Inc., Hercules, CA, USA). The real time quantitative PCR were performed on a 7900 HT fast real-time PCR system (Applied Biosystems, Foster, CA, USA) using 2 µl cDNA, 7 µl diethylpyrocarbonate water, 10 µl SsoFast™ Probes Supermix (BIO-RAD Inc., Hercules, CA, USA) and 1 µl primer of TaqMan Gene Expression Assay. Forty cycles of amplification were performed. Cycle threshold (Ct) values were determined using SDS 2.3 software. The gene of interest was normalized to the Ct value of the endogenous reference gene, 18s rRNA, using the ΔCt method described by Pfaffl [81]. The primer/probe sequences are listed in Table 4.

**Table 4:** Quantitative real time RT-PCR primers.

Gene of Interest	Forward Primer	Reverse Primer
***18s***	GAGAAACGGC TACCACATCC	GGACACTCAG CTAAGAGCATCG
***IL1ß***	AAGGCTCTCCACCTCCTCA	TTGATCCCTAAGGTCACAGGTATCT
***TNFα***	CCTACTGCACTTCGAGGTTATCG	GGCCAGAGGGTTGA
***has1***	CTCGGCGACTCGGTGGACTAC	GGGGACCACTGATGCAGGACA
***has2***	AGCAGCCCATTGAACCAGGGACTTG	AGGGTCGGTGGCGGGCAGTTTCCAAAAC
***has3***	CCTACTTTGGCTGTGTGAAA	AGGCTGGACATATAGAGAAG
***hyal1***	CAGTGCCCTAGGTGGACC	CACCCGATCCTTGAGTGAG
***hyal2***	CGGTATAGGTCTCCCAGTTCTG	CAGGCGCAGTATGAATTTGAG

Forward and reverse primers used for quantitative real time RT-PCR.

### Myeloperoxidase and N-acetylglucosaminidase Activities

Myeloperoxidase (MPO) and N-acetyl-ß-glucosaminiadase (NAG) activities served as markers of lung neutrophil and macrophage contents respectively, and were determined as previously described [82]. Lung tissue was weighed for normalization of data. For MPO activity, 25 µl of sonicated samples or 4.15 - 83 µU of MPO standards (Sigma, St. Louis, MO) were loaded in 96-well plates. Sixty microliters 0.1M K_2_PO_4_, 20 µl 0.5% H_2_O_2,_ and 20 µl 1.25 mg/ml O-diansidine (ICN Biomedicals, Irvine, CA) were added to each well. After incubation at room temperature for 15 minutes, the reaction was stopped by the addition of 20 µl 1% sodium azide. The change in absorbance at 450 nm over the 15 minutes provides an index of MPO activity and correlates with neutrophil content. For NAG activity, 20 µl of sonicated sample or 25–400 µU of ß-N-acetylglucosaminidase A standard (Sigma, St. Louis, MO) was loaded in 96-well plates. Ten microliters 0.1% Triton X and 20 µl 15 mM p-nitrophenyl N-acetyl-β-D-glucosaminide (Sigma, St. Louis, MO) were added to each well. After incubation at 37°C for 30 minutes, 200 µl 0.2 M sodium carbonate was added to each well to stop the reaction. The change in absorbance at 405 nm was measured as an index of NAG activity and correlates with macrophage content.

### ELISA for TNFα and IL1ß

Serum TNFα and IL1ß concentrations were determined using commercially available ELISA kits (R&D Systems) as per manufacturer’s instructions.

### In vitro Analysis of HA Fragmentation by Peroxynitrite

In order to confirm superoxide and peroxynitrite fragmentation of HA, we exposed Healon™, a clinical grade HMW HA product, to various conditions and then examined HA size using gel electrophoresis and staining with *StainsAll*. HMW HA (Healon™, 1×10^6^ Da) was exposed to 100 µM 3-morpholinosydnonimine (SIN-1), a compound that spontaneously decomposes at pH 7.4 to release NO and superoxide, thereby generating peroxynitrite. HA was also exposed to SIN-1 in the presence of superoxide dismutase (600 U/ml) to decrease superoxide and peroxynitrite. Exposure to PAPANOATE (300 µM), a pure NO donor that does not generate either superoxide or peroxynitrite was used as a control. In addition, Healon™ was treated with either *Streptomyces* hyaluronidase (1 U/ml) at 60°C for 2 hours or sonication for 2 minutes, methods known to fragment HA. In addition to Hyalose HMW and LMW HA markers, Healon™ was used as a marker of HMW HA and HA of molecular size 200 kDa (ICN) was used as a marker for LMW HA.

### HA Oligosaccharide Stimulation of RAW264.7 Cells

The murine macrophage cell line RAW264.7 was stimulated by various concentrations of HA6, a six sugar oligosaccharide obtained from Seikagaku Corporation (Tokyo, Japan), which was shown to be free of endotoxin, protein and nucleic acid. Cells (1×10^6^) were exposed to 10 µg/ml HA6 for 24 hours. The supernatant was spun to clear cells and stored at −80°C for TNFα determination. The cells were harvested and IL1ß concentrations determined in equal amounts of protein lysate.

### Statistical Analysis

At least 7 animals were included in each group. All animal physiology data are presented as mean ± SD. For *in vitro* studies, experiments were repeated at least three times and representative data are shown. Results are presented as mean ± SEM. Percent control data were calculated by using the mean of all control animals. Differences among groups were analyzed using one-way Analysis of Variance (ANOVA). When statistically significant differences were found (p<0.05), individual comparisons were made using the Bonferroni/Dunn tests.
